# Differential expression of skeletal muscle genes following administration of clenbuterol to exercised horses

**DOI:** 10.1186/s12864-016-2945-2

**Published:** 2016-08-09

**Authors:** Heather K. Knych, Linda M. Harrison, Stacy J. Steinmetz, Nadira Chouicha, Phil H. Kass

**Affiliations:** 1 K.L. Maddy Equine Analytical Chemistry Laboratory, School of Veterinary Medicine, University of California, Davis, USA; 2 Department of Veterinary Molecular Biosciences, School of Veterinary Medicine, University of California, Davis, USA; 3 Willow Oak Equine, Woodland, CA USA; 4 Department of Population Health and Reproduction, School of Veterinary Medicine, University of California, Davis, USA

**Keywords:** Horse, Clenbuterol, Gene expression, TLDA, Microarray, β2 agonist

## Abstract

**Background:**

Clenbuterol, a beta2-adrenergic receptor agonist, is used therapeutically to treat respiratory conditions in the horse. However, by virtue of its mechanism of action it has been suggested that clenbuterol may also have repartitioning affects in horses and as such the potential to affect performance. Clenbuterol decreases the percent fat and increases fat-free mass following high dose administration in combination with intense exercise in horses. In the current study, microarray analysis and real-time PCR were used to study the temporal effects of low and high dose chronic clenbuterol administration on differential gene expression of several skeletal muscle myosin heavy chains, genes involved in lipid metabolism and the β2-adrenergic receptor. The effect of clenbuterol administration on differential gene expression has not been previously reported in the horse, therefore the primary objective of the current study was to describe clenbuterol-induced temporal changes in gene expression following chronic oral administration of clenbuterol at both high and low doses.

**Results:**

Steady state clenbuterol concentrations were achieved at approximately 50 h post administration of the first dose for the low dose regimen and at approximately 18–19 days (10 days post administration of 3.2 μg/kg) for the escalating dosing regimen. Following chronic administration of the low dose (0.8 μg/kg BID) of clenbuterol, a total of 114 genes were differentially expressed, however, none of these changes were found to be significant following FDR adjustment of the *p*-values. A total of 7,093 genes were differentially expressed with 3,623 genes up regulated and 3,470 genes down regulated following chronic high dose administration. Of the genes selected for further study by real-time PCR, down-regulation of genes encoding myosin heavy chains 2 and 7, steroyl CoA desaturase and the β2-adrenergic receptor were noted. For most genes, expression levels returned towards baseline levels following cessation of drug administration.

**Conclusion:**

This study showed no evidence of modified gene expression following chronic low dose administration of clenbuterol to horses. However, following chronic administration of high doses of clenbuterol alterations were noted in transcripts encoding various myosin heavy chains, lipid metabolizing enzymes and the β2-adrenergic receptor.

## Background

Clenbuterol is a β2-adrenergic receptor agonist that is labeled for use in the management of horses affected with airway obstruction. Clenbuterol, a moderately selective β2-adrenergic receptor agonist, causes relaxation of airway smooth muscle by binding to the receptor and activating adenylyl cyclase. Activation of adenylyl cyclase leads to an increase in intracellular concentrations of cyclic adenosine monophosphate (cAMP) and ultimately activation of protein kinase A (PKA). In the airways this inhibits smooth muscle contraction by opening K^+^ channels and down-regulating myosin light chain kinase activity [1].

β2-adrenergic agonists are also known to promote lipolysis and increase muscle mass in many species [2–8]. This repartitioning effect and improvement in muscle growth efficiency has led to the addition of β2-adrenergic agonists such as ractopamine and zilpaterol to feed products for production animals. While not approved for use as a growth-promoting agent, by virtue of its mechanism of action, clenbuterol when administered at high doses has been shown to have similar repartitioning effects in many species [2, 4–7]. Since clenbuterol is an approved therapeutic medication that is commonly used in racehorses, regulators have expressed concern regarding its potential to alter body composition in such a way as to affect performance. Supporting this concern are reports of decreases in the percent fat and increases in fat-free mass following high dose clenbuterol administration (2.4 μg/kg BID) in horses when combined with intense exercise [5]. However, it should be noted that although changes in body mass have been documented in many species, performance is not necessarily improved and in some cases may be hindered [4, 9, 10].

Increases in myofibrillar and structural proteins following clenbuterol administration have been well documented for several years [2–8]. More recently investigators have used gene expression analysis techniques including DNA microarray and qRT-PCR to elucidate the underlying mechanism for these changes [11, 12]. To date the molecular effects of clenbuterol administration on gene expression have been described in production animals and laboratory animal species [11, 12]. Spurlock and colleagues [11] demonstrated changes in the mRNA abundance of multiple genes associated with myogenic differentiation in mice following clenbuterol administration. Increases in expression levels of lipid metabolism related genes were demonstrated in pigs following clenbuterol administration [12]. Investigators speculated that one of these genes, *apoR*, might be critical in reducing fat accumulation [12].

While the effect of high dose clenbuterol on skeletal muscle fiber thickness in the horse has been assessed [6], there are no studies assessing the effects of clenbuterol at the molecular level in this species. Therefore, in the current study we chose to use DNA technology to assess the changes in skeletal muscle gene expression following high dose administration of clenbuterol to horses. Furthermore, studies describing muscle hypertrophy in horses have utilized high doses of the compound [5, 6] and although clenbuterol is labeled for use at doses as high as 3.2 μg/kg twice a day (BID), a more common therapeutic dosing regimen in performance horses in the United States is chronic low dose (0.8 μg/kg, BID) administration. Therefore, the primary objective of the current study was to describe clenbuterol-induced temporal changes in gene expression following chronic oral administration of clenbuterol at both high and low doses. We hypothesized that clenbuterol would affect skeletal muscle gene expression (increased or decreased depending on the gene), relative to baseline levels, in the horse in a dose dependent manner.

## Methods

### Animals, clenbuterol treatment and sampling

Twenty-eight healthy, exercised adult Thoroughbred horses (11 mares and 17 geldings; weight range of 470.7 ± 25.0 kg; age range of 2–6 years) were studied. Horses used in this study were a combination of University and client owned horses. This study was approved by the Institutional Animal Care and Use Committee (University and client owned horses) and the Clinical Trials Committee of the University of California, Davis (client owned horses). For client owned horses, written consent was obtained for participation in the study. Prior to and throughout the course of the study, horses were exercised five days a week. The general exercise protocol was meant to simulate the strenuous exercise of race training. The exercise regimen for these horses consists of three days per week on an Equineciser (Centaur Horse Walkers Inc, Mira Loma, CA, USA) (5 min at walk; 30 min trot; 5 min walk) and two days per week on a high speed treadmill (Mustang 2200, Graber AG, Switzerland; Day 1: 5 min @1.6 m/s; 5 min @ 4 m/s; 5 min @ 7 m/s; 5 min @ 1.6 m/s all at 6 % incline. Day 2: 3 min @ 1.6 m/s; 4 min @ 4.0 m/s; 2 min @ 7.0 m/s; 2 min @ 11.0 m/s and 5 min @1.6 m/s all at 3 % incline). All horses were subject to regular fitness testing, including weekly heart rate measurements and calculation of V_200_ (running velocity that elicited a heart rate 200 bpm) and monthly measurements of end run plasma lactate concentrations, as a means by which to ensure that the fitness level of the horses used in this study were as comparable as possible to the average racehorse.

Prior to commencement of the study, all horses were determined healthy and free of cardiovascular diseases by physical examination, complete blood count and a serum biochemistry panel that included aspartate aminotransferase, creatinine phosphokinase, alkaline phosphatase, total bilirubin, sorbital dehydrogenase, blood urea nitrogen and creatinine. Horses did not receive any other medications for at least two weeks prior to conducting this study.

Twenty-two of the horses received 0.8 μg/kg clenbuterol (Ventipulmin®, Boehringer Ingelheim Vetmedica Inc, St Joseph, MO) PO BID for 30 days and an additional 6 horses received the escalating dosing protocol as described on the manufacturer label for non-responders (0.8 μg/kg, BID × 3 days; 1.6 μg/kg, BID × 3 days; 2.4 μg/kg, BID × 3 days; 3.2 μg/kg, BID for 21 days).

Blood samples were collected at time 0 and at 15, 30, and 45 min, and 1, 1.5, 2, 2.5, 3, 3.5, 4, 5, 6, 8 and 12 h post administration of the first dose and last dose and 18, 24, 36, 48, 60, 72, 84, 96, 108 and 120 h post administration of the last dose. Additionally blood samples were collected every 12 h (immediately prior to administration of each dose (trough concentrations)) throughout the 30-day dosing period. Blood samples were collected by direct venipuncture into EDTA blood tubes (Kendall/Tyco Healthcare, Mansfield MA) and were centrifuged at 3000 × g for 10 min. Plasma was immediately transferred into storage cryovials (Phenix Research Products, Chandler, NC) and stored at −20 ° C until analysis. Drug concentrations were measured by Liquid Chromatography tandem Mass Spectrometry as described previously [13]. 

Muscle biopsy samples were collected one day prior to administration of the first dose of clenbuterol. Additional samples were collected at 48 h and on days 7, 14, and 28 post administration of the first dose. A final sample was collected one-week post administration of the final dose (35 days post administration of the first dose). Muscle biopsies were collected at the same time on each sampling occasion which was approximately 3–4 h post feeding. Horses were not exercised on the day that muscle biopsy samples were collected. For sample collection, horses were sedated with a combination of xylazine (Lloyd Inc, Shenandoah, IA) and butorphanol (Zoetis, Florham Park, NJ). Lidocaine (Aspen Veterinary Resources Ltd, Liberty MO) was administered subcutaneously over the gluteus muscle. A small area over the superficial gluteal muscle was aseptically prepped and a Bergstrom biopsy needle (Dixons Surgical Instruments, Wickford, Essex) used to collect a small sample (approximately 1 g of tissue). The tissue was transferred to a cryovial containing RNA*later* (Qiagen Inc, Valencia, CA) and stored at −20 ° C until processed.

### RNA extraction and quality assessment

Muscle biopsy samples were placed in 600 μl of lysis buffer, contaning 2-β- mercaptoethanol (Qiagen Inc, Valencia, CA). The tissue sample was then transferred to MagNa Green Beads tubes (Roche Diagnostics, Mannhein, Germany). Homogenization was performed using a MagNaLyse (Roche Diagnostics, Mannhein, Germany) at 6000 rpm for 30 s followed by a 1.0 min cool down period. Homogenization intervals were continued until pieces of muscle were no longer visible (≤3 times). Total RNA was purified using an miRNeasy Mini kit (RNeasy Mini, Qiagen Inc, Valencia, CA) and following the manufacturer’s instructions. Total RNA integrity was assessed using the Experion Automated Electrophoresis System (Bio-rad, Hercules, CA). Only RNA samples with RIN ≥ 8 and 260/280 ratios between 1.7 and 2.1 were used [14, 15].

### Microarray analysis

Equine specific microarrays (EquGene-1.0-st; Affymetrix, Santa Clara, CA), containing 504,603 probes representing 30,559 well-characterized genes were used.

To reduce biological noise as a result of genetic variability, each horse (5 horses for low dose administration and 4 horses for the escalating dosing regimen) was analyzed separately and served as their own control for comparison of baseline samples to day 14 (low dose administration) or day 28 (escalating dose regimen). Five biological replicates per time point were tested. Purified total RNA (5 μg) was used for cDNA synthesis in accordance with the Ambion® WT Expression assay kit (Affymetrix, Santa Clara, CA) manufacturer’s protocol. In vitro transcription was used to incorporate biotin labels using the GeneChip® WT Terminal Labeling system (Affymetrix, Santa Clara, CA) and samples hybridized to the Equine microarray. Arrays were washed and stained on a Fluidics Station 450 (Affymetrix, Santa Clara, CA) and scanned on a GeneChip Scanner 3000 (Affymetrix, Santa Clara, CA) in accordance with manufacturer’s protocols.

The microarrays were evaluated for differential gene expression using Transcriptome Analysis Console (TAC) and for hybridization quality control using Expression Console Software (Affymetrix, Santa Clara, CA). In brief, a total of five Cell Intensity Files were generated per time point, uploaded and normalized under the following conditions: PM (perfect match)-only as a PM intensity adjustment and the Robust Multichip Analysis (RMA) quantification method. For evaluation of the assays performance the number of differentially expressed (DE) genes detected between baseline and day 14 (low dose administration) or day 28 (escalating dose regimen) were assessed. Based on the TAC software user’s manual, genes with mean transformed ratios significantly less than −2 and larger than +2 were considered significantly regulated. Significant DE genes were selected by filtering the genes using an ANOVA (*p* value < 0.05). A Pearson’s correlation coefficient was used to calculate linear dependence between time point and baseline samples to evaluate the correlation coefficient, where 1 was a positive correlation and 0 was no correlation. For each probeset, expression at day 14 (low dose administration) or day 28 (escalating dose regimen) was compared to expression at baseline in the same horse using a paired *t*-test. Fold changes and their confidence intervals were calculated by exponentiating (base 2) the mean within-horse difference in expression for each gene and the associated t confidence intervals. *P*-values were adjusted for multiple testing using the False Discovery Rate (FDR) method of Benjamini and Hochberg [16]. Analyses were conducted using the statistical software environment R, version 3.0.2 (R Core Team, 2013). Gene Ontology biological process of differently expressed genes of interest was performed using the most recent Database for Annotation, Visualization and Integrated Discovery (DAVID) 6.8 Beta (https://david-d.ncifcrf.gov/) with the Entrez Gene ID as the primary identifier and *Equus Caballus* as the species.

### Quantification of mRNAs using Taqman low density arrays (TLDA)

Genes that showed significant changes in expression (greatest fold change and statistical significance) following microarray analysis as well as those that have previously been reported to be affected by clenbuterol administration, were chosen as candidate genes for further study. Microarray data were validated by measuring the levels of specific mRNA in muscle biopsy samples at each time point post clenbuterol administration vs. baseline via Taqman Low Density Arrays (TLDA; Affymetrix, Santa Clara, CA) which are pre-loaded 384 well RT-PCR microfluidic cards.

RNA was diluted to a concentration of 2 ng/μl for cDNA conversion using the QuantiTect Reverse Transcription Kit (Qiagen Inc., Valencia, CA). cDNA was then combined with Taqman Universal Mastermix (Affymetrix, Santa Clara, CA) at a final concentration of 2X and then 100ul of each sample loaded onto the TLDA card.

Primers for candidate genes were designed and manufactured by Life Technologies (Affymetrix, Santa Clara, CA). Reference or “housekeeping” genes were used to evaluate the effect of RNA integrity on the array and qRT-PCR performance. Based on stability across all samples studied (TaqMan Protocol [17]) equine beta 2 microglobulin (*β2M*) was used as the endogenous control gene to normalize the qRT-PCR data while human 18 s (hs18s) was used as an internal manufacturing control. Each sample was run in quadruplicate with 10 candidate genes per card. The TLDA cards were then run on a QuantStudio™ 12 K Flex Software v1.2.2 (Affymetrix, Santa Clara, CA). Analysis was performed using ExpressionSuite Software 1.0.3 using Singleplex analysis (Affymetrix, Santa Clara, CA).

Statistical analyses using commercially available software (Stata/IC 13.1, StataCorp LP, College Station, TX) were performed to assess significant differences in expression (fold change) between baseline and each time point as well as between the different time points post clenbuterol treatment. Data was analyzed using a mixed effects analysis of variance, with the horse treated as a random effect, and with time as fixed effects. Post-hoc comparisons were performed with a Bonferonni multiple-comparison adjustment to preserve a nominal significance level of 0.05.

## Results

### Plasma clenbuterol concentrations

**Fig. 1:**
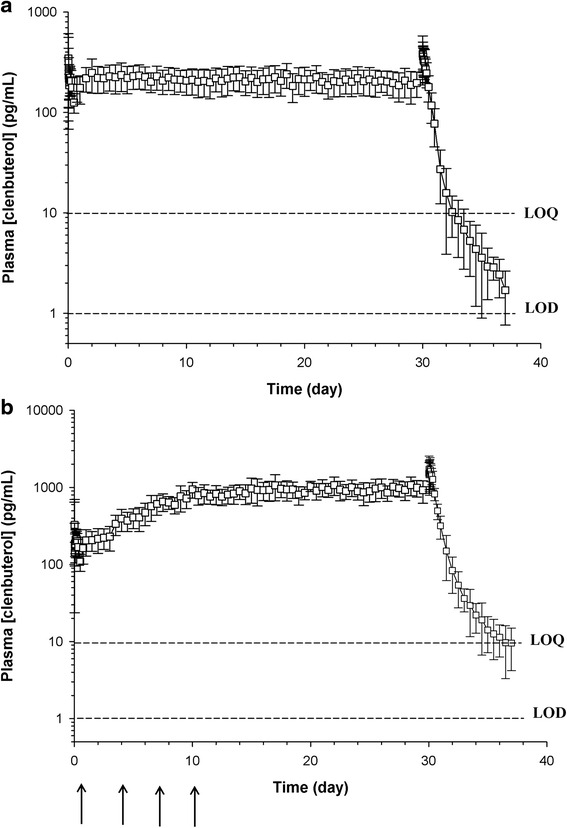
Mean ± SD plasma clenbuterol concentrations vs time following chronic (**a**) low dose and (**b**) high dose administration of (**a**) 0.8 μg/kg Ventipulmin® BID for 30 days to 22 racing fit Thoroughbred horses and (**b**) Ventipulmin® (0.8 μg/kg, BID for 3 days, 1.6 μg/kg, BID for 3 days, 2.4 μg/kg, BID for 3 days and 3.2 μg/kg, BID for 21 days) to 6 racing fit Thoroughbred horses. Arrows indicate administration of a new dose

The average clenbuterol plasma concentration over time-curves following chronic low dose and escalating dose administrations are depicted in Fig. 1a, b, respectively. Clenbuterol plasma concentrations were below detectable levels by day 7 post administration of the final dose in all 22 horses studied at the low dose and below the LOQ of the assay by day 7 in 5/6 horses receiving the escalating dosing protocol. The terminal elimination half-life was 10.4 ± 4.4 h (mean ± SD) following chronic low dose administration and 46.2 ± 8.37 h (mean ± SD) following administration of the last dose (3.2 μg/kg) of the escalating dosing protocol. Steady state concentrations were achieved at approximately 50 h post administration of the first dose for the low dose regimen and at approximately 18–19 days (10 days post administration of 3.2 μg/kg) for the escalating dosing regimen.

### CDNA microarray analysis

**Fig. 2:**
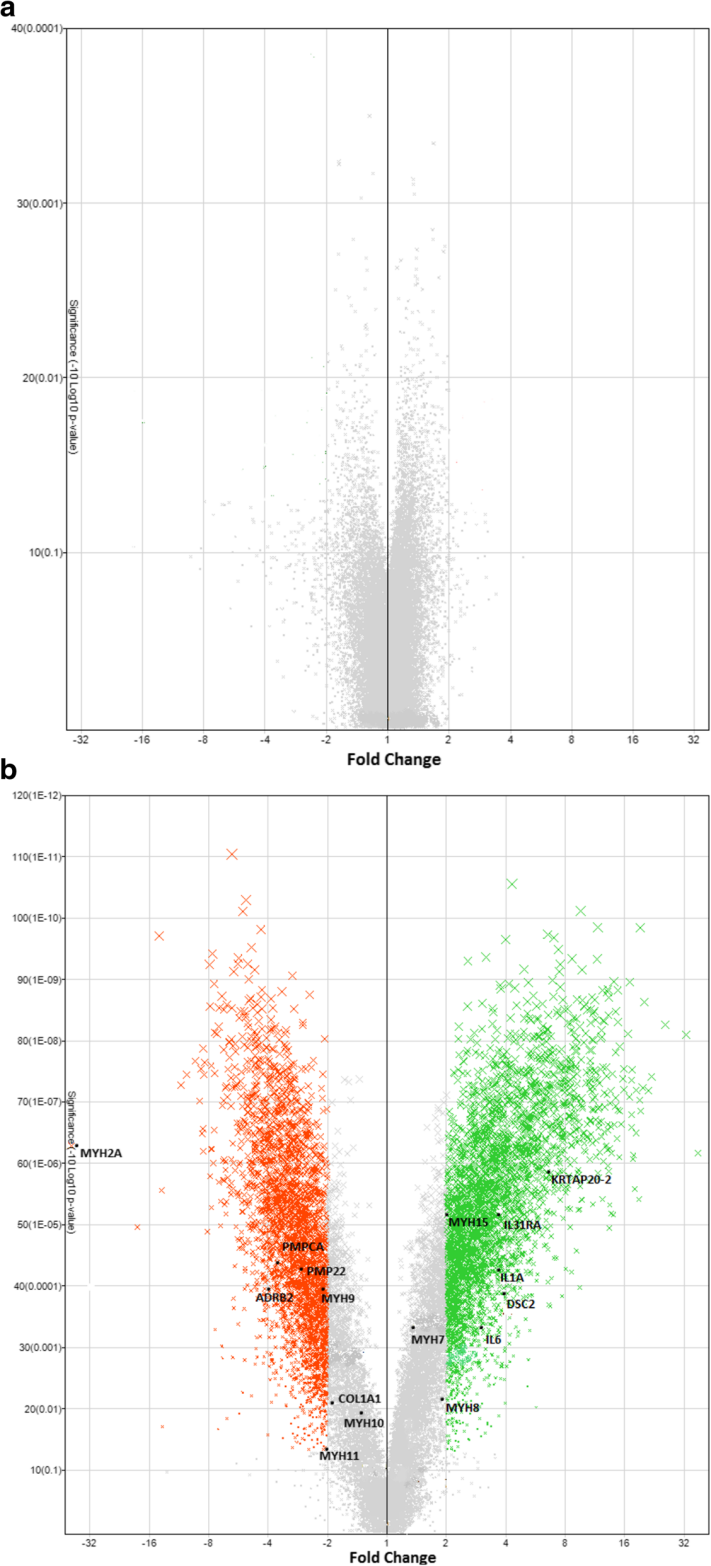
Volcano plot of differentially abundant transcripts at 2 weeks following oral administration of 0.8 μg/kg Ventipulmin® BID for 30 days to 22 racing fit Thoroughbred horses and (b) 4 weeks following oral administration of Ventipulmin® (0.8 μg/kg, BID for 3 days, 1.6 μg/kg, BID for 3 days, 2.4 μg/kg, BID for 3 days and 3.2 μg/kg, BID for 21 days) to 6 racing fit Thoroughbred horses. Red and green dots represent genes that are significantly down-regulated and up-regulated, respectively

Equine specific microarrays containing expression profiling of 25,923 well-characterized genes were utilized. The data has been deposited in the NCBI’s Gene Expression Omnibus (GEO) [18] and are accessible through GEO series accession number GSE79559. Baseline samples collected the day prior to drug administration were compared to those collected on day 14 (low dose administration) or day 28 (escalating dose regimen). Following chronic administration of the low dose (0.8 μg/kg BID) of clenbuterol, a total of 114 genes were DE, however, none of these changes were found to be significant following FDR adjustment of the *p*-values. The volcano plot of differentially abundant transcripts generated from microarray analysis is depicted in Fig. 2a.

Following administration of the escalating dosing protocol increases in expression levels of a number of genes were noted. The volcano plot of differentially abundant transcripts generated from microarray analysis is depicted in Fig. 2b. A total of 7,093 genes were DE with 3,623 genes up regulated and 3,470 genes down regulated. Differentially expressed genes were divided into groups according to their Gene Ontology Biological Process descriptions (Table 1). A total of 537 unique genes were identified and classified. The largest number of genes were regulation of cellular process related (247). Forty-three genes were actin-filament-based process related, 28 involved in muscle system processes and 10 related to striated muscle contraction.

**Table 1:** Differentially expressed genes of interest (*p* < 0.05) categorized according to the Gene Ontology biological process terms

Gene ontology biological process category	Number of genes
Regulation of cellular process	247
Localization	177
Proteolysis	172
Regulation of development	125
Death	117
Morphogenesis	116
Cell Adhesion	99
Biological Adhesion	99
Intracellular Transport	95
Homeostasis	74
Membrane Organization	72
Cell Differentiation	71
Cell Communication	58
Actin-Filament Based Process	43
Muscle System Process	28
Response Stimulus	20
Striated Muscle Contraction	10

A total of 537 unique gene identifiers were identified and classified in categories with a minimum of 6 members

Sixteen selected DE genes are listed in Table 2. Based on the results of the microarray analysis (greatest fold change and statistical significance) and previously reported effects of clenbuterol on skeletal muscle and lipid metabolism, 13 genes were selected for further analysis to quantitate the change in expression levels, relative to baseline, at several time points during the dosing period and following termination of clenbuterol administration.

**Table 2:** Selected differentially expressed genes utilizing an equine specific microarray following administration of an escalating dosing regimen (0.8 μg/kg, BID × 3 days; 1.6 μg/kg, BID × 3 days; 2.4 μg/kg, BID × 3 days; 3.2 μg/kg, BID for 21 days) to 6 exercised Thoroughbred research horses. For microarray analysis, baseline (pre-dose) transcript levels were compared to transcript levels on day 28

Gene symbol	Gene name	Fold change	*P* value (FDR corrected)
*MYH2A*	Myosin Heavy Chain 2A, skeletal muscle	−30.5	<0.001
*MYH7*	Myosin Heavy Chain 7, cardiac and skeletal	1.39	0.002
*MYH8*	Myosin Heavy Chain 8, skeletal muscle	1.86	0.014
*MYH9*	Myosin Heavy Chain 9, non-muscle	−2.60	0.001
*MYH10*	Myosin Heavy Chain 10, non-muscle	−1.31	0.028
*MYH11*	Myosin Heavy Chain 11, smooth muscle	−2.24	0.033
*MYH15*	Myosin Heavy Chain 15	2.10	<0.001
*ADRB2*	Adrenoreceptor, beta 2	−4.0	0.001
*COL1A1*	Collagen, type 1, alpha 1	−1.8	0.019
*IL1A*	Interleukin 1, alpha	3.42	<0.001
*IL6*	Interleukin 6	3.38	0.002
*IL31RA*	Interleukin 31, receptor A	2.77	<0.001
*DSC2*	Desmocollin 2	2.95	0.001
*PMP22*	Peripheral myelin protein 22	−2.5	<0.001
*PMPCA*	Peptidase alpha	−2.64	<0.001
*KRTAP20-2*	Keratin associate protein 20-2	7.32	<0.001

### Quantification of mRNAs using Taqman low density arrays (TLDA)

**Fig. 3:**
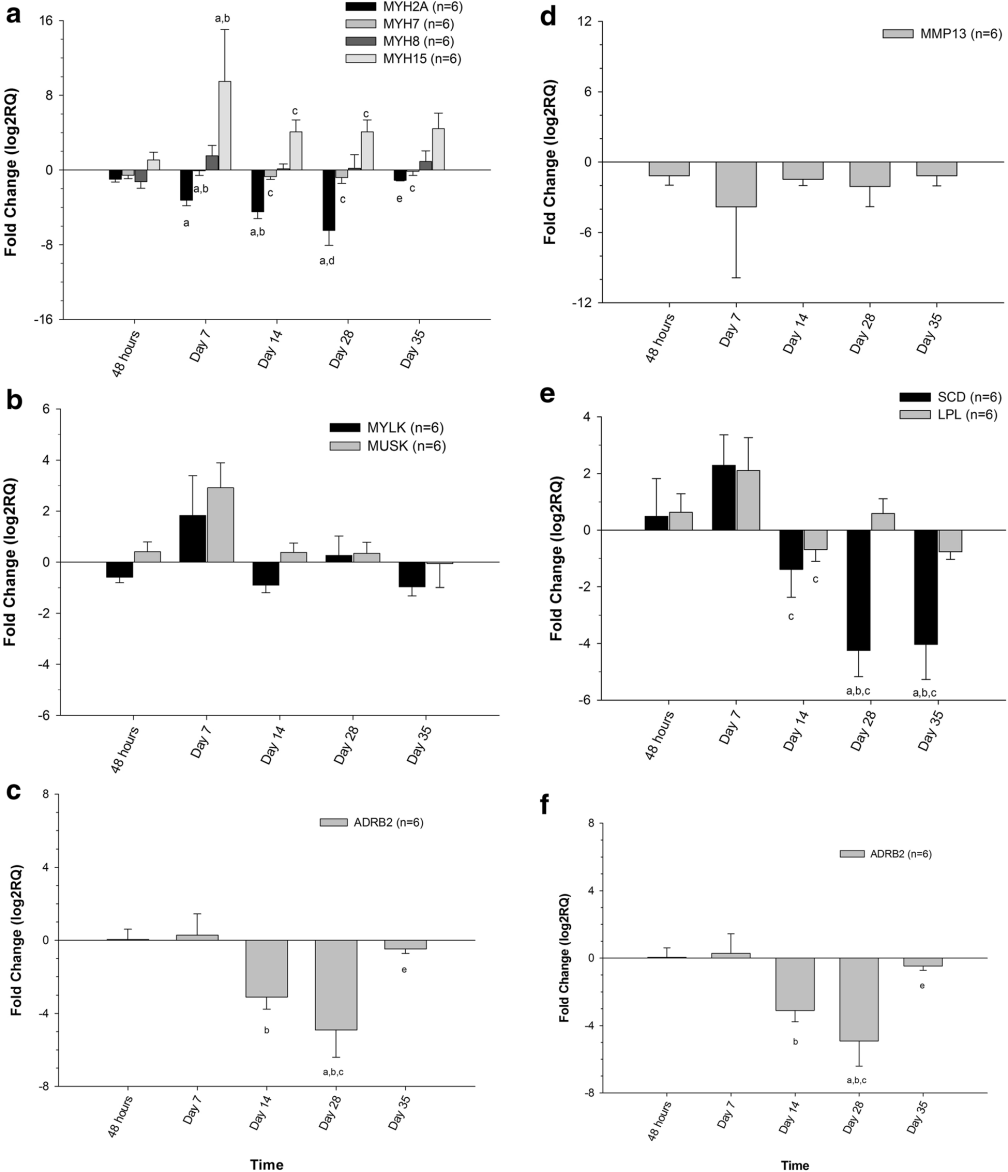
Mean relative expression (compared to baseline) of **a** myosin heavy chains (*MYH2A, MYH7, MYH8, MYH15*), **b** myosin light chain kinase (*MYLK*) and skeletal muscle receptor tyrosine kinase (*MUSK*), **c** collagen type 1A1 (*COL1A1*), collagen type 1A2 (*COL1A2*) and collagen type 3A1 (*COL3A1*), **d** matrix metalloproteinase 13 (*MMP13*), **e** steroyl CoA desaturase (*SCD*) and lipoprotein lipase (*LPL*) and **f** β2-adrenergic receptor ((*β2R*) in skeletal muscle following oral administration of clenbuterol at the high dose protocol (Ventipulmin®;0.8 μg/kg, BID for 3 days, 1.6 μg/kg, BID for 3 days, 2.4 μg/kg, BID for 3 days and 3.2 μg/kg, BID for 21 days) to 6 racing fit Thoroughbred horses. ^a^represents changes that are significantly different (*p* <0.05) from baseline (pre-treatment), ^b^represents changes that are significantly different (*p* <0.05) from 48 h, ^c^represents changes that are significantly different (*p* <0.05) from day 7, ^d^represents changes that are significantly different (*p* <0.05) from day 14, ^e^represents changes that are significantly different (*p* <0.05) from day 28, ^f^represents changes that are significantly different (*p* <0.05) from day 35

Genes selected for TLDA analysis included: β2-adrenergic receptor (*β2R*), myosin heavy chain 2 (*MYHC2A*), myosin heavy chain 7 (*MYH7*), myosin heavy chain 8 (*MYH8*), myosin heavy chain 15 (*MYHC15*), myosin light chain kinase (*MYLK*), muscle skeletal receptor tyrosine kinase (*MUSK*), collagen type 3A1 (*COL3A1*), collagen type 1A1 (*COL1A1*), collagen type 1A2 (*COL1A2*), matrix metalloproteinase 13 (*MMP13*), steroyl CoA desaturase (*SCD*) and lipoprotein lipase (*LPL*). Expression levels, relative to baseline, at each time during and post clenbuterol administration for genes analyzed using TLDA are depicted in Fig. 3.

Of the 13 genes selected for TLDA analysis, 7 showed a similar significant change (up or down-regulation) in expression as that demonstrated on microarray analysis. While changes in expression were detected for the remaining 6 genes (*MYH8, MYLK, MUSK, COL3A1, COL1A2, MMP13 and LPL*) these were not deemed statistically significant.

*MYH2A* was significantly down-regulated, compared to baseline, on days 7, 14 and 28 post clenbuterol administration with transcript levels returning towards normal on day 35 (7 days post administration of the final dose). *MYH7* was also down-regulated starting on day 7 and continuing through day 35. On day 7, transcript was significantly different from baseline and 48 h and subsequent samples were significantly different from day 7 levels. On day 7, *MYH15* was significantly different from baseline and 48 h transcript levels and on days 14 and 28, it was significantly different from day 7. The only collagen gene affected following clenbuterol administration was *COL1A2* on days 14 (significantly different from baseline transcript levels) and 28 (significantly different from day 14). Although not significant, *SCD* transcript levels first increased relative to baseline (48 h and day 7). Starting with the day 14 sample and continuing through the final time point (day 35), *SCD* transcripts were significantly down-regulated compared to baseline. The β2 adrenergic receptor was up-regulated, although not significantly so at the first two time points (48 h and day 7). Significant down-regulation of the *β2R* gene was noted on days 14 (compared to 48 h), 28 (compared to baseline, 48 h and day 7) and 35 (compared to day 28).

## Discussion

Clenbuterol administration has been associated with changes in body composition including alterations in lipid metabolism and changes in skeletal muscle fibers [2–8]. Numerous studies have shown that these drug-induced alterations are a result of changes at the molecular level, namely changes in mRNA expression [11, 12]. Alterations in body composition, including a significant shift in the myosin heavy chain protein profile [6] and alterations in plasma leptin and adipopnectin concentrations [7] have been reported following clenbuterol administration to horses. However, to the best of the authors’ knowledge, this is the first report to describe temporal alterations in mRNA expression following clenbuterol administration to horses, using 2 commonly used dosing regimens.

As a means of providing a global overview of genes affected by clenbuterol administration, microarray analysis, comparing mRNA levels prior to drug administration to the levels at 2 weeks (low dose administration) or 4 weeks (escalating dose regimen) post drug administration was used. No significant changes in gene expression were noted following administration of the chronic low dose protocol (0.8 ug/kg BID × 30 days). Conversely, following high dose chronic administration, significant changes were noted in genes coding for select myosin heavy chain isoforms, proteins involved in fatty acid metabolism and the β2 adrenergic receptor. The remainder of this discussion pertains to the high dose (escalating dosing) regimen.

### Skeletal muscle fibers

Myosins are actin-based motor proteins that function in the generation of mechanical force [19] and are responsible for producing contraction in muscle cells. There are a number of isoforms of myosin heavy chains and fiber type is dependent on the isoform that is present. Myosin heavy chains are encoded by a highly conserved multi-gene family and include isoforms such as 2A, 2B and 2X [19]. The *MYH2B* gene is not expressed in equine skeletal muscle [20]. Myosin heavy chain 7 is another member of the myosin family found primarily in cardiac but also in skeletal muscle [21] and encoded by the *MYH7* gene.

Alterations in the skeletal muscle fiber profile have been described following administration of β2 adrenergic agonists, including clenbuterol and ractopamine [11, 12, 22, 23]. At the protein level, this most commonly involves a shift from the MYH1 to the MYH2A and 2X fiber type [24–26]. In the current study, there was not a significant change in either *MYH1* or *MYH2X* gene expression on microarray analysis, therefore, these genes were not selected for further study. In a study similar to the currently reported one, ractopamine was administered to pigs, yielding similar results with respect to *MYH1* expression [22]. An apparent lack of agreement between expression of *MYH* transcripts and protein isoforms has been described previously in horses and was attributed to the presence of hybrid fibers [27]. Additionally, other investigators have theorized that fibers that have more than one MYH protein and only one transcript could be a result of fibers converting to the type corresponding to the expressed transcript [28–30].

A decrease in MYH2A protein levels has been demonstrated in horses following administration of clenbuterol [6]. Similarly, in the current study, *MYH2A* gene expression was significantly down regulated (relative to baseline). This was first observed on day 7 and continued throughout the dosing period with maximum effects on expression occurring on 28. This was also the time at which steady state clenbuterol concentrations post the final dose escalation, were achieved, suggesting a dose dependent effect. *MYH2A* levels were not significantly different from baseline at the last time point tested, which was 5 days after termination of dosing, further supporting a dose dependent effect.

### Genes involved in lipid metabolism

SCD is responsible for converting fatty acids into mono-unsaturated fatty acids in mammalian adipocytes [31]. Up-regulation of the *SCD* gene in skeletal muscle has been associated with abnormal lipid partitioning and obesity in humans and lower levels of SCD with a leaner body type [32]. β2 adrenergic agonists have demonstrated effects on lipid metabolism [12], including down-regulation of *SCD*. This has been demonstrated in pigs following 4 weeks of treatment with ractopamine (25 mg/kg BID; [12]). In the current study, *SCD* mRNA expression decreased starting on day 14 and continued until 7 days post clenbuterol administration (day 35). This time course is highly suggestive of a dose-dependent effect as no effects on *SCD* were observed following chronic low dose administration (0.8 ug/kg BID × 30 days). Furthermore, following administration of the escalating non-responder dosing regimen, significant effects were not observed until day 14, after significant drug accumulation had occurred.

### β2- adrenergic receptor

Chronic administration of β2-adrenergic agonists has been associated with down-regulation of the β2-adrenergic receptor in humans [33] and horses [34]. In conjunction with this down-regulation, a number of investigators have noted a decrease in *β2R* mRNA [34]. Decreased levels of *β2R* mRNA has been reported in fast twitch muscle fibers in rats following clenbuterol administration [33, 35] and in pigs following long-term ractopamine administration [22]. In the current study, *β2R* mRNA levels were unaffected following low dose clenbuterol administration, however, following administration of the escalating dosing protocol, mRNA levels decreased significantly by days 14 and 28. Abraham and colleagues [34] demonstrated a down-regulation of β2-adrenergic receptors following chronic clenbuterol administration and theorized that this provided an explanation for variable sensitivity of horses with chronic obstructive pulmonary disease to β2-adrenergic agonists as well as the development of tolerance with long term treatment. Similarly in the current study, decreased *β2R* mRNA expression as a result of high dose chronic clenbuterol administration may lead to down-regulation of β2-adrenergic receptors and offer an explanation for the decreased expression of *MYH, MYLK, MUSK, ACD* and *LPL*. By day 35 (7 days post termination of drug administration), *β2R* levels had returned towards baseline levels, presumably due to decreasing concentrations of clenbuterol.

## Conclusion

In the current study, we describe changes in gene expression in skeletal muscle following chronic administration of clenbuterol to horses. Changes in gene expression were not noted following administration of therapeutic low doses, however, following chronic administration of high doses of clenbuterol alterations were noted in transcripts encoding various myosin heavy chains, lipid metabolizing enzymes and the *β2R*.

## Abbreviations

β2R, β2-adrenergic receptor; BID, Twice a day; cAMP, cyclic adenosine monophosphate; COL1A1, collagen type 1A1; COL1A2, collagen type 1A2; COL3A1, collagen type 3A1; DE, differentially expressed; FDR, false discovery rate; GEO, gene expression omnibus; LPL, lipoprotein lipase (LPL); MMP13, matrix metalloproteinase 13; MUSK, muscle skeletal receptor tyrosine kinase; MYH7, myosin heavy chain 7; MYH8, myosin heavy chain 8; MYHC15, myosin heavy chain 15; MYHC2A, myosin heavy chain 2; MYLK, myosin light chain kinase; PKA; protein kinase A; RMA, robust multichip analysis; SCD, steroyl CoA desaturase; TLDA, taqman low density arrays
